# Evaluating Non-Invasive Computer Vision-Based Quantification of Neonatal Movement as a Marker of Development in Preterm Infants: A Pilot Study

**DOI:** 10.3390/healthcare13131577

**Published:** 2025-07-01

**Authors:** Janet Pigueiras-del-Real, Lionel C. Gontard, Isabel Benavente-Fernández, Syed Taimoor Hussain, Syed Adil Hussain, Simón P. Lubián-López, Angel Ruiz-Zafra

**Affiliations:** 1 Department of Condensed Matter Physics, University of Cádiz, 11510 Cádiz, Spain; lionel.cervera@uca.es; 2 IMEYMAT, University of Cádiz, 11510 Cádiz, Spain; 3 Division of Neonatology, Department of Paediatrics, Puerta del Mar University Hospital, 11019 Cádiz, Spain; isabel.benavente@uca.es (I.B.-F.); simon.lubian@uca.es (S.P.L.-L.); 4 Research Unit, Biomedical Research and Innovation Institute of Cádiz (INiBICA), 11019 Cádiz, Spain; 5 Area of Paediatrics, Department of Child and Mother Health and Radiology, Medical School, University of Cádiz, 11003 Cádiz, Spain; 6 PolitoBIOMed Lab, Department of Mechanical and Aerospace Engineering, Politecnico di Torino, 10129 Turin, Italy; taimoor.shah@polito.it; 7 Department of Research and Development (R&D), GPI SpA, 38123 Trento, Italy; syedadilhussain.shah@gpi.it; 8 Department of Software Engineering, University of Granada, 18071 Granada, Spain

**Keywords:** preterm, neonatal intensive care unit, movement quantification, computer vision, pose tracking, non-invasive, computational methods

## Abstract

**Background**: Traditional neonatal assessments rely on anthropometric measures such as weight, body size, and head circumference. However, recent studies suggest that objective movement quantification may serve as a complementary clinical indicator of development in preterm infants. **Methods**: This study evaluates non-invasive computer vision-based quantification of neonatal movement using contactless pose tracking based on computer vision. We analyzed approximately 800,000 postural data points from ten preterm infants to identify reliable algorithms, optimal recording duration, and whether whole-body or regional tracking is sufficient. **Results**: Our findings show that 30 s video segments are adequate for consistent motion quantification. Optical flow methods produced inconsistent results, while distance-based algorithms—particularly Chebyshev and Minkowski—offered greater stability, with coefficients of variation of 5.46% and 6.40% in whole-body analysis. Additionally, Minkowski and Mahalanobis metrics applied to the lower body yielded results similar to full-body tracking, with minimal differences of 0.89% and 1%. **Conclusions**: The results demonstrate that neonatal movement can be quantified objectively and without physical contact using computer vision techniques and reliable computational methods. This approach may serve as a complementary clinical indicator of neonatal progression, alongside conventional measures such as weight and size, with applications in continuous monitoring and early clinical decision-making for preterm infants.

## 1. Introduction

Preterm infants, defined as those born before the 37th week of gestation, face higher risks of various health complications due to their underdeveloped organs and systems [1]. These infants are more vulnerable to respiratory conditions such as bronchopulmonary dysplasia, as well as cardiovascular problems, infections, and neurodevelopmental disorders [2].

Their premature birth profoundly affects their growth and development, requiring specialized care and continuous monitoring [3]. As a result, preterm infants are often admitted to the Neonatal Intensive Care Unit (NICU), where healthcare professionals provide the critical support needed to address respiratory, cardiac, and neurodevelopmental challenges they may face during this vulnerable stage [4].

Recent advances in technology have opened new opportunities to improve neonatal care, particularly through the application of computer vision techniques in clinical evaluations [5]. Computer vision-based methods allow for automated, objective, and scalable analyses of medical imaging and video data, making them an increasingly valuable tool in neonatal health monitoring [6]. By leveraging these techniques, healthcare professionals can gain deeper insights into infant development, such as the detection of early indicators of neurodevelopmental disorders with greater accuracy and efficiency than traditional observational methods [7,8].

When determining a preterm infant’s readiness for hospital discharge, several key factors are evaluated to ensure the baby is developing appropriately and can thrive outside the controlled environment of the neonatal unit [9]. These factors encompass various physiological and developmental benchmarks that collectively provide a comprehensive view of the infant’s health and progress [10].

Key clinical indicators for assessing discharge readiness in preterm infants include weight gain, which should follow intrauterine growth patterns (typically 15–20 g/kg/day) and reflects adequate nutrition and metabolic function [11,12]; head circumference growth, which serves as a proxy for brain development and should progress steadily alongside other growth parameters [13,14]; and vital sign stability, demonstrated by consistent heart rate, respiratory rate, and temperature regulation, as well as independence from supplemental oxygen and intravenous nutrition [12,15,16,17]. Feeding efficiency is also essential, with preterm infants expected to feed effectively by breast or bottle without respiratory support, ensuring sufficient nutritional intake [10,12,17]. Neurological status is typically evaluated through imaging, motor assessments, and sensory screenings to rule out early developmental issues [18,19].

These factors are medically considered the key indicators for determining the evaluation of preterm infants. On the other hand, recent research has also emphasized the growing importance of neonatal movement as a key indicator of an infant’s health and development [20,21]. This emerging perspective suggests that, in addition to traditional clinical measures (size, weight, etc.), evaluating both the quality and quantity of neonatal movements can offer a more detailed understanding of the infant’s neurological development and provide valuable insights into their readiness for discharge [22,23].

To date, the evaluation of movement in preterm infants has traditionally been a manual process, relying on the expertise of neonatologists or professionals. This manual validation is typically carried out in accordance with Novak’s guide, in which the GMA (General Movements Assessment) by Prechtl is recognized as one of the three key tools for the early detection of cerebral palsy. This assessment enables clinicians to evaluate the infant’s neurological development based on the observation of spontaneous movements [24,25].

The quantification of movement has shown promise as a valuable tool for identifying early signs of neurodevelopmental challenges, especially in preterm infants [26,27]. For instance, studies have linked specific movement patterns, such as poor repertoire movements and cramped-synchronized movements, to adverse outcomes, correlating them with structural brain abnormalities and an increased risk of developmental delays [22,23]. Therefore, the quantification of movement in neonates, along with its proper interpretation, could aid in the early detection of abnormalities, enabling timely intervention [28,29]. In this context, movement quantity could serve as a crucial indicator of a preterm infant’s progress. When considered alongside other key metrics—such as weight, size, and vital sign stabilization—it can provide valuable support for neonatologists in assessing the infant’s overall development and prognosis.

On the other hand, the significance of normal movement patterns extends beyond neuromotor development. Consistent and varied movements are associated with better developmental outcomes, whereas reduced movement raises concerns about potential brain injuries or other health issues [30]. In this context, movement quantification has been shown to support clinical interventions and improve patient outcomes [22,23].

However, no standardized method or objective quantifier is currently available for use as a medical indicator. As an alternative to the traditional assessment of a preterm infant’s movement quantity by medical personnel, computer vision is emerging as a promising solution for precisely quantifying these movements, enabling automated and real-time analysis of neonatal motor activity [7,31].

Computer vision technologies, often combined with artificial intelligence, allow for the extraction of body landmarks from video frames to analyze movement patterns and identify specific actions [32,33]. Tools such as YOLO^®^, OpenPose^®^, and MediaPipe^®^ generate positional data that can be used to reconstruct body pose and assess motion. While these methods have been applied in various populations—such as athletes and elderly individuals in rehabilitation [34,35]—their application to preterm infants remains limited. Furthermore, identifying the most appropriate computational approach for quantifying movement in this vulnerable group continues to pose significant challenges.

This study explores the non-invasive computer vision-based quantification of neonatal movement, aiming to provide neonatologists and healthcare professionals with an objective and dependable indicator to support the assessment of neonatal development.

To achieve this, this research explores various general-purpose approaches for non-invasive, computer vision-based quantification of neonatal movement. While not originally developed for this specific task, these techniques show potential for application in this context. The study evaluates their effectiveness in accurately measuring movement using body posture data. Additionally, it investigates the optimal time frame for observing or recording a preterm infant to ensure precise movement quantification. Another key aspect of this research is determining whether a whole-body analysis is necessary or if focusing on specific body regions—such as the trunk, upper or lower limbs, or arms—provides sufficient data for a reliable and meaningful assessment of movement.

Although the cohort comprises a limited number of neonates (n = 10), this study draws upon a high-resolution dataset containing over 800,000 posture-related entries. As such, the findings—despite their quantitative richness—should be regarded as preliminary and intended to illustrate potential trends rather than establish conclusive evidence.

The main contributions of our work are as follows:

- Since this study has direct applications in real-time computer vision systems, it has been determined that movement quantity is proportional to observation duration. Consequently, relatively short data intervals (e.g., 30 s recordings) are sufficient for reliably estimating movement.
- Although various computational methods can be applied, the results of the study indicate that optical flow-based approaches are not the most suitable for a population group such as preterm infants. Instead, more traditional distance-based methods, such as Minkowski or Chebyshev distance, provide more reliable results while requiring fewer computational resources. This makes them more practical and reliable for application in preterm infants.
- Given the number of body landmarks analyzed, this study has shown that using the entire body consistently yields reliable results. However, combining a specific computational method with a particular body region (e.g., the lower body) produces comparable results, with an error margin below 10%. For example, Minkowski and Mahalanobis distances yield similar results in estimating the amount of movement when using either the whole body or only the lower body, with a movement difference of 0.89% and 1%, respectively.
- The findings of this study have potential applications in neonatology, contributing to the advancement of non-invasive, automated movement monitoring as a practical tool for tracking neonatal development over time. The ability to quantify movement could serve as a valuable factor in assessing motor development, aiding in the early detection of potential motor or neurological impairments and supporting timely interventions. Additionally, movement quantification can function as an indicator of neonatal progression, providing an objective measure to track developmental trends over time.

The remainder of this article is structured as follows: Section 2 details the population group, the dataset, and the scientific methodology used. Section 3 illustrates and describes the results and outcomes of the study. Section 4 offers a comprehensive discussion of these results, and highlights the significance of the research presented. Finally, Section 5 summarizes our conclusions, highlighting the study’s contributions to the integration of the computer vision and computational methods in neonatal healthcare. Additionally, it outlines future research directions and potential areas for further exploration.

## 2. Materials and Methods

### 2.1. Study Design and Research Questions

This study is designed to systematically explore three key research questions that define its objectives and methodological approach. These questions guide the assessment of different movement quantification methods to determine the most effective methods, the identification of the optimal observation period, and the selection of the most relevant body regions for neonatal motor assessment. By addressing these aspects, the study aims to improve the accuracy, efficiency, and clinical relevance of automated movement analysis in neonatal care.

To achieve these objectives, the study focuses on the following research questions:

- RQ1: What time interval is optimal, or at least minimal, for determining a quantity of movement that can be used medically as an indicator of a premature infant’s evolution over time?
- RQ2: What method could be applied to numerically estimate this quantity of movement, and which methods are suitable for premature infants?
- RQ3: When determining the quantity of movement, is it necessary to use the whole body, or is it sufficient to rely on data from specific limbs (e.g., left arm, right arm, torso) or groups of limbs (upper body or lower body)?

Regarding RQ1, the quantification of movement is associated with a time interval. In this context, and from a medical perspective, it would be desirable to determine how long it is necessary to observe a premature baby for to obtain an objective measure of how much they have moved during that time. This can then be used to determine, in comparison to previous measurements, whether the baby is moving more or less, serving as an indicator of their development, similar to how weight or size is used. For this study, we have defined four time intervals (in seconds): 30, 60, 120, and 180.

The question RQ2 arises regarding which method is most suitable for determining the quantity of movement within a set of frames, where the (*x, y, z*) coordinates of several body points (landmarks) and timestamps are available. Although the most direct approach, which has been used with other population groups, is the use of Euclidean distance, there are numerous alternative methods in the literature that could be applicable to the specific case of premature neonates [36]. In this study, a total of nine methods were applied for movement quantification. Seven of these are distance-based or kinematic approaches: Euclidean distance, Manhattan distance, Chebyshev distance, Minkowski distance, Mahalanobis distance, Differential Acceleration, and Angular Displacement. Additionally, two optical flow methods, the Lucas–Kanade method, and the Farnebäck method, were considered as potential alternatives for movement quantification.

Finally, it has been observed that premature neonates show favorable development when the amount of movement in certain limbs changes: for example, transitioning from more random movements in the lower limbs to more deliberate movements, smoother head movements, or, in general, increased body movement over time from the date of admission to the NICU to the date of hospital discharge [37,38]. In this study, to address RQ3, we analyzed the movement of the study subjects in 9 different groups: the whole body, head movements only, left arm, right arm, torso, left leg, right leg, upper body (from the waist up), and lower body (from the waist down).

With these functionalities, for each subject of the study (preterm infant), structured JSON files were generated for each time interval (RQ1), each body part (RQ2), and each motion quantification method (RQ3) containing the following information:

- Values: a list of values of variable length (depending on the time interval), where each value represents the total quantification of applying the selected motion quantification method to the dataset within that time interval.
- Min value: the minimum value from the list of values.
- Max value: the maximum value from the list of values.
- Average (avg): the arithmetic mean of the list of values.
- Standard deviation (std): the standard deviation of the mean with respect to the list of values, which is the variability in movement across frames.
- Median: the median of the list of values, which is the central tendency of movement.

In this way, for 10 subjects, with 4 time intervals, across 9 body regions, and applying a total of 9 methods, the study generates 3240 structured files as a result.

**Figure 1:**
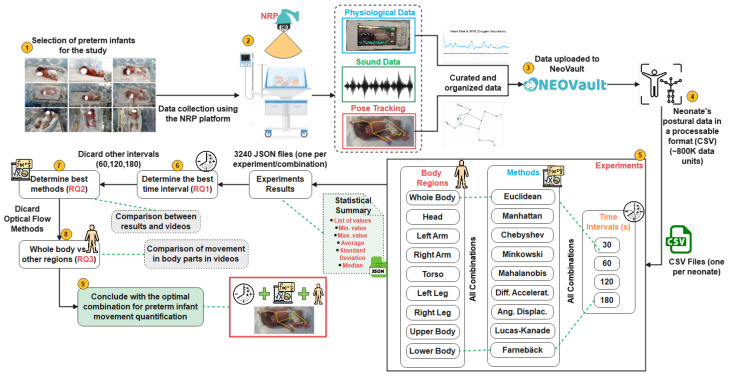
Flowchart and methodological overview.

Building on these research questions, the flowchart in Figure 1 presents a structured overview of the study methodology, outlining the sequential process from data collection to the comparative evaluation of movement quantification methods, ultimately leading to the identification of the most effective approach. The following sections provide a detailed description of each step in this workflow.

### 2.2. Data Collection and Preprocessing

The data were automatically gathered using the Neonates Recording Platform (NRP), a system specifically designed, deployed, and validated for seamless, organized, and efficient data collection in hospital settings [39]. NRP allows the automatic scheduling of trials, during which data are collected from various sources: audio (both ambient and within the incubator), physiological parameters (heart rate, oxygen saturation), custom labels (defined by medical staff), study metadata (start date, end date, data summary, etc.), and, finally, postural data. All data are collected automatically and stored in a well-structured folder for each neonate, where they are saved in CSV format, curated, and cleaned for subsequent processing.

**Figure 2:**
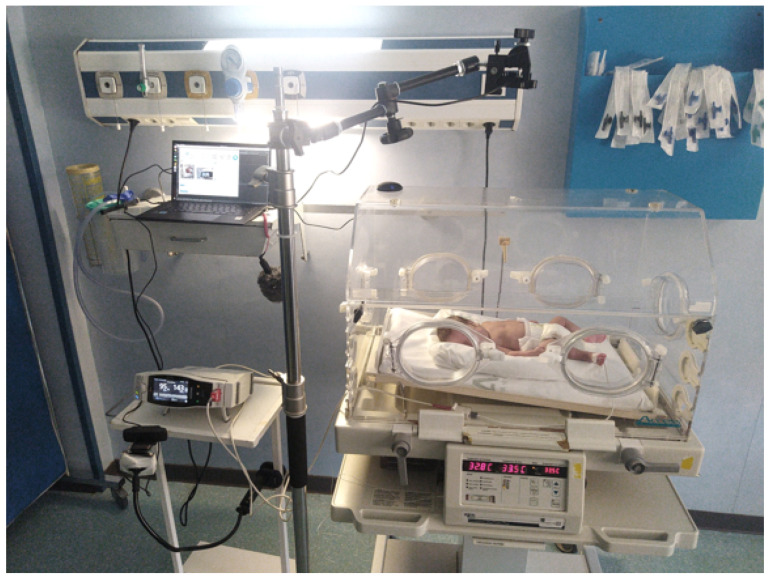

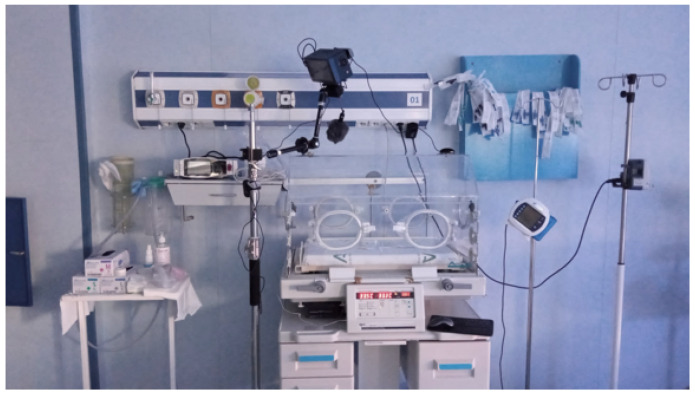
Data collection setup in the hospital using NRP [39].

Figure 2 illustrates the data collection setup in the hospital, using NRP, as previously described in [39]. This figure has been reproduced under a Creative Commons Attribution 4.0 International (CC BY 4.0) license, allowing its unrestricted use with appropriate citation.

**Figure 3:**
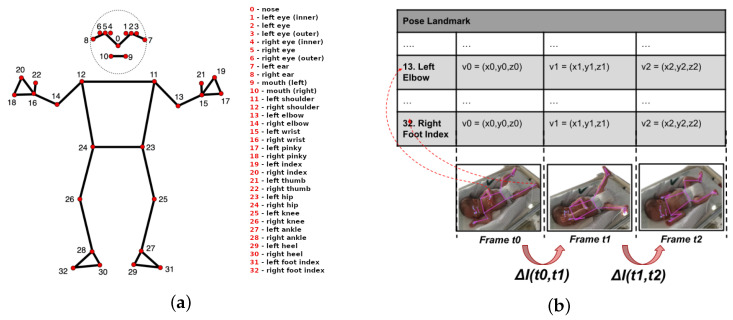
Human body landmark and inter-frame motion estimation approach. (**a**) Landmarks on the human body. (**b**) Inter-frame motion changes.

Regarding the postural data, which are the focus of the study described in this article, 33 landmarks were collected by the NRP platform for each frame of video using the Google AI-Edge MediaPipe library (see Figure 3a) (https://ai.google.dev/edge/mediapipe/solutions/vision/pose_landmarker (accessed on 27 June 2025)).

These data are stored in a structured format (comma-separated values—CSV), which includes 33 columns for positional data corresponding to the 33 landmarks, along with an additional column for the timestamp. Each entry in the file contains data for every landmark, represented as a tuple (x,y,z) and the timestamp in milliseconds (Unix Timestamp). The *x* coordinate indicates the horizontal position relative to the input image, normalized to a range between 0 and 1, where 0 corresponds to the left edge of the image and 1 to the right edge. Similarly, the *y* coordinate represents the vertical position, following the same normalized range, where 0 corresponds to the top edge and 1 to the bottom edge. The *z* coordinate represents the relative depth, with negative values indicating that the landmark is closer to the camera and positive values indicating it is farther away. These tuple values are dimensionless and can be mapped to specific positions in the image (for example, to visualize a pose skeleton) by scaling them according to the image’s width and height. This setup enables the generation of time series that accurately represent a neonate’s movements.

### 2.3. Dataset

All collected data were preprocessed by NRP, which cleaned and prepared the postural, physiological, and medical data of the neonates datasets before they were uploaded to NeoVault. This dedicated data hub provides structured access to the cleaned data through a user-friendly website and a RESTful API, following the OpenAPI standard [40]. NeoVault facilitates easy downloading of the data in a processable format (CSV), streamlining further analysis. Additionally, all raw postural data are hosted on GitHub (https://github.com/bihut/neovault-database (accessed on 27 June 2025)). For this study, approximately 800,000 postural data points collected from study subjects over time were utilized.

It is worth noting that the dataset used in this study aligns with the principles of the General Data Protection Regulation (GDPR). As mentioned earlier, the data were collected using the Neonates Recording Platform (NRP), which allows for both video and landmark-only recording. This means that it is not necessary to store any video frames of the neonates; instead, only a set of anonymized numerical coordinates (landmarks) associated with a non-identifiable user ID is retained. This approach ensures that no biometric images are stored and significantly reduces privacy risks while still enabling meaningful motion analysis.

### 2.4. Motion Quantification Methods

#### 2.4.1. Overview

The objective of this study is to determine the method that most closely approximates the actual amount of motion using the postural data collected from the study subjects.

There are several well-documented approaches in the literature for estimating movement from a video (or set of frames). Some of the most common methods include estimating object displacement across frames [41], comparing pixel blocks [42], identifying reference points and tracking their changes over time, measuring changes in intensity or texture (e.g., using histograms) [43], and performing spectral analysis via transformations (e.g., Fourier transforms) [44]. One of the most widely used approaches, however, is based on approximating movement through the absolute difference in pixel values between frames [45,46], where the total amount of motion will be determined based on the weighted difference between the (x,y,z) values corresponding to each frame over time (see Figure 3b).

The motion difference could be represented as $\Delta I(t_{1},t_{2})$, and the total motion difference for a set of frames in a video over a given duration could be defined as:

$$\Delta I_{\mathrm{total}}=\sum_{i=0}^{n-1}\Delta I\left(t_{i},t_{i+1}\right)$$

where

- $t_{0},t_{1},\ldots ,t_{n}$ represent the sequence of frames.
- $\Delta I(t_{i},t_{i+1})$ denotes the motion difference between consecutive frames $t_{i}$ and $t_{i+1}$.
- $\Delta I_{\mathrm{total}}$ is the cumulative motion difference over the entire sequence.

Thus, the variation in landmark positions between frames over time provides a basis for estimating movement quantification. This study focuses on movement quantification using body landmarks rather than relying on pixel-based motion estimation, which avoids limitations related to lighting variability and background changes.

#### 2.4.2. Methods

To analyze movement dynamics, nine motion quantification methods were implemented. These are categorized into the following:

- Distance-based methods or kinematic approaches: Euclidean distance, Manhattan distance, Chebyshev distance, Minkowski distance, Mahalanobis distance, Differential Acceleration, and angular displacement.
- Optical flow methods: Lucas–Kanade and Farnebäck.

Each of these methods is briefly described below.

##### Euclidean Distance

The Euclidean distance measures the straight-line distance between two points in a three-dimensional space [47]. It is the most commonly used metric for quantifying spatial movement. The formula for Euclidean distance between two points $\left(x_{1},y_{1},z_{1}\right)$ and $\left(x_{2},y_{2},z_{2}\right)$ is given by:

$$d_{\mathrm{Euclidean}}=\sqrt{{\left(x_{2}-x_{1}\right)}^{2}+{\left(y_{2}-y_{1}\right)}^{2}+{\left(z_{2}-z_{1}\right)}^{2}}$$

##### Manhattan Distance

The Manhattan distance, also known as taxicab distance, calculates the distance between two points as the sum of the absolute differences between their coordinates [48]. Unlike the Euclidean distance, it only considers movements along the grid, similar to how a taxi might move in a city with strict grid-based streets. The formula is:

$$d_{\mathrm{Manhattan}}=|x_{2}-x_{1}\left|+\right|y_{2}-y_{1}\left|+\right|z_{2}-z_{1}|$$

This method is useful when considering movements restricted to axes-based directions rather than diagonals.

##### Chebyshev Distance

The Chebyshev distance considers only the maximum difference along any coordinate axis between two points [49]. It is the longest of the distances calculated in each dimension, making it useful for situations where only one dimension is relevant. The formula is,

$$d_{\mathrm{Chebyshev}}=\max\{|x_{2}-x_{1}\left|,\right|y_{2}-y_{1}\left|,\right|z_{2}-z_{1}\left|\right\}$$

This method is suitable when prioritizing movement in a specific direction.

##### Minkowski Distance

The Minkowski distance is a generalization of both Euclidean and Manhattan distances, depending on a parameter *p*. For p=2, it reduces to the Euclidean distance, and for p=1, it reduces to the Manhattan distance [50]. The formula is:

$$d_{\mathrm{Minkowski}}={\left(|x_{2}-x_{1}{|}^{p}+|y_{2}-y_{1}{|}^{p}+{\left|z_{2}-z_{1}\right|}^{p}\right)}^{1/p}$$

This flexibility allows for adjusting the trade-off between spatial directions.

##### Mahalanobis Distance

The Mahalanobis distance is a measure of the distance between two points A and B in a multivariate space, taking into account the correlations between variables [51]. In a three-dimensional space, where $A=(x_{1},y_{1},z_{1})$ and $B=(x_{2},y_{2},z_{2})$, the Mahalanobis distance is defined as:

(1) $$d_{M}\left(A,B\right)=\sqrt{{\left(A-B\right)}^{T}S^{-1}\left(A-B\right)}$$

where *S* is the covariance matrix of the dataset, and $S^{-1}$ is its inverse.

First, define the difference vector:

(2) $$D=\left[\begin{array}{c} x_{2}-x_{1}\\ y_{2}-y_{1}\\ z_{2}-z_{1} \end{array}\right].$$

The covariance matrix *S* is given by:

(3) $$S=\left[\begin{array}{ccc} \sigma_{x}^{2} & \sigma_{xy} & \sigma_{xz}\\ \sigma_{yx} & \sigma_{y}^{2} & \sigma_{yz}\\ \sigma_{zx} & \sigma_{zy} & \sigma_{z}^{2} \end{array}\right],$$

where

- $\sigma_{x}^{2},\sigma_{y}^{2},\sigma_{z}^{2}$ are the variances of the variables *x*, *y*, and *z*.
- $\sigma_{xy},\sigma_{xz},\sigma_{yz}$ are the covariances between the respective variables.

Finally, the Mahalanobis distance is computed as:

(4) $$d_{M}=\sqrt{D^{T}S^{-1}D}.$$

##### Differential Acceleration

This metric quantifies the total acceleration difference across multiple frames in a dataset, providing a comprehensive measure of dynamic changes in motion [52]. The Differential Acceleration method evaluates the variation in acceleration for tracked landmarks across consecutive frames in a dataset of three-dimensional points. To achieve this, positional data are extracted for each landmark over three consecutive frames: the previous, current, and next frame. Based on this information, velocity is computed for each spatial dimension using the frame rate as a scaling factor. The velocity at a given frame is determined by the difference in position between successive frames, scaled by:

(5) $$V_{i-1,i}^{\left(d\right)}=\left(P_{i}^{\left(d\right)}-P_{i-1}^{\left(d\right)}\right)\cdot fps,$$

(6) $$V_{i,i+1}^{\left(d\right)}=\left(P_{i+1}^{\left(d\right)}-P_{i}^{\left(d\right)}\right)\cdot fps.$$

The acceleration difference is then computed as the variation in velocity between consecutive frames:

(7) $$A_{diff}^{\left(d\right)}=V_{i,i+1}^{\left(d\right)}-V_{i-1,i}^{\left(d\right)}.$$

Finally, to obtain a comprehensive measure of motion dynamics, the absolute values of these acceleration differences are summed across all dimensions and all landmarks in the dataset:

(8) $$DA=\sum_{i=1}^{N}\sum_{d\in x,y,z}\left|A_{diff}^{\left(d\right)}\right|,$$

where N represents the total number of landmarks. This approach provides a robust framework for quantifying motion variations by analyzing frame-to-frame acceleration changes.

##### Angular Displacement

Angular displacement quantifies changes in rotational motion over time [53]. This method measures the variation in the angular position of tracked landmarks between consecutive frames in a dataset. Specifically, for three consecutive frames, the angular positions of each landmark are extracted in a three-dimensional space. The angular displacement is then computed using the angle between vectors representing the landmark positions across different frames. Mathematically, it is determined using the dot product formula:

(9) $$\cos\theta =\frac{a\cdot b}{\left|a||b\right|}.$$

By applying the inverse cosine function, we obtain:

(10) $$\theta =\arccos\left(\frac{a\cdot b}{\left|a||b\right|}\right).$$

The total angular displacement is obtained by summing up the absolute angular variations for all landmarks throughout the dataset:

(11) $$DA_{angular}=\sum_{i=1}^{N}\sum \left|\theta_{i}\right|,$$

where N represents the total number of landmarks. This method provides a robust mechanism for tracking orientation changes in motion analysis.

##### Lucas–Kanade Method

The Lucas–Kanade method is a differential approach to estimating optical flow by assuming that motion remains constant within a small spatial window [54]. Given a set of tracked points in three-dimensional space, the motion estimation steps are as follows.

First, The spatial derivatives $I_{x}$, $I_{y}$, and $I_{z}$ and the temporal derivative $I_{t}$ are computed using central finite differences:

(12) $$I_{x}=\frac{P_{i+1}^{\left(x\right)}-P_{i-1}^{\left(x\right)}}{2},\;I_{y}=\frac{P_{i+1}^{\left(y\right)}-P_{i-1}^{\left(y\right)}}{2},\;I_{z}=\frac{P_{i+1}^{\left(z\right)}-P_{i-1}^{\left(z\right)}}{2},$$

(13) $$I_{t}=P_{i+1}-P_{i}.$$

The system of equations for motion estimation is given by:

(14) Av=b,

where A represents the matrix of spatial gradients and the temporal gradient contributions. The velocity components are then estimated using:

(15) $$v={\left(A^{T}A\right)}^{-1}A^{T}b.$$

Finally, the total motion magnitude is obtained as:

(16) ||v||=|v|.

This formulation provides a compact and efficient method for estimating 3D motion dynamics by analyzing frame-to-frame positional changes in tracked landmarks.

##### Farnebäck Method

The Farnebäck method estimates motion by computing optical flow using a set of assumptions about how pixels are displaced between frames [55].

In this implementation, 3D landmarks are converted into synthetic images, and grayscale transformations are applied to facilitate motion estimation. Given two consecutive grayscale images, the Farnebäck method estimates displacement vectors by approximating local intensity variations with quadratic polynomials:

(17) $$I\left(x,y\right)\approx x^{T}Ax+b^{T}x+c,$$

where A is a symmetric matrix encoding the second-order derivatives, b represents the first-order derivatives, and c is a constant term. By computing the transformation of these polynomials between frames, the method derives a dense optical flow field. The magnitude of the estimated optical flow is computed as:

(18) $$M=\sum_{x,y}\left|F\left(x,y\right)\right|,$$

where M represents the total estimated movement across all pixels. This implementation enhances the analysis of motion dynamics in tracked landmarks by summing the total optical flow over the dataset.

### 2.5. Experimental Setup

To conduct the study, all of these approaches (Euclidean distance, Manhattan distance, etc.) were implemented in Python (https://www.python.org, accessed on 27 June 2025) using various libraries, such as Pandas (https://pandas.pydata.org/ (accessed on 27 June 2025)), OpenCV (https://opencv.org/ (accessed on 27 June 2025)), NumPy (https://numpy.org/) (accessed on 27 June 2025), and SciPy (https://scipy.org/ (accessed on 27 June 2025)). Many of these methods are easy to implement, such as the Euclidean distance. Others, while more comprehensive, are natively supported by libraries. A notable example is the Farnebäck method, which is provided in the OpenCV library through the calcOpticalFlowFarneback() function (https://docs.opencv.org/3.4/de/d9e/classcv_1_1FarnebackOpticalFlow.html (accessed on 27 June 2025)).

The generated files are analyzed using another Python script that leverages the previously mentioned libraries and Matplotlib for graphical representation (https://matplotlib.org/ (accessed on 27 June 2025)). This visualization helps assess the amount of movement over time, determined by each method across different time intervals and subjects of the study, aiming to identify which option is the most valid and accurate for quantitatively determining movement.

## 3. Results

The results presented in the following sections are based on a cohort of 10 preterm neonates who were monitored throughout 2023 during their stay in the Neonatal Intensive Care Unit (NICU). The study was approved by the Ethics Committee of Puerta del Mar University Hospital (Cádiz, Spain) in December 2022 under registration number 125.22, and written informed consent was obtained from the parents prior to recruitment.

Data collection was carried out on days when no medical procedures interfered and only with parental approval to avoid disrupting family routines.

Table 1 summarizes the main clinical characteristics of the study subjects and the duration of each recording session, providing relevant context for the performance evaluation of the proposed motion quantification methods.

After conducting a comprehensive analysis of the positional data of the neonates involved in this study using nine computational methods for movement quantification, we now aim to address the initial research questions (RQ1, RQ2, and RQ3) outlined in Methods (Section 2).

The results presented in this section focus on three key aspects: (1) determining the optimal time interval for neonatal movement quantification, (2) identifying the most effective methods for movement estimation, and (3) evaluating whether whole-body movement can be approximated by targeted limb data.

### 3.1. RQ1-Determining the Optimal Time Interval for Quantifying Neonatal Movement

**Figure 4:**
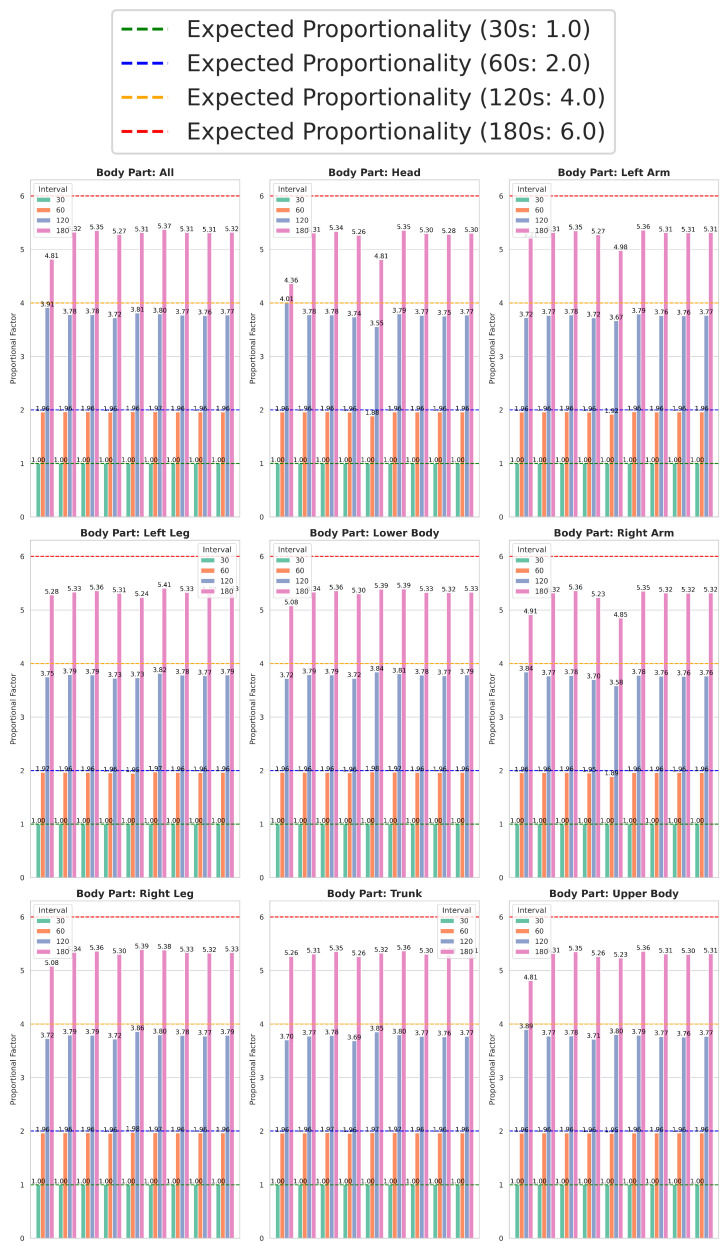
Standardized comparison across computational methods, whole body, body regions, and time intervals.

Figure 4 illustrates the differences in average movement quantity across computational methods, whole-body movement, and individual body parts over four time intervals (30, 60, 120, and 180 s). The data reveal a clear proportional trend: for a given computational method and body part, the total movement detected tends to approximately double at 60 s, quadruple at 120 s, and increase sixfold at 180 s relative to the 30 s interval. This pattern suggests that extending the interval generally results in a predictable scaling of movement values, following this proportional tendency.

Given this proportionality, the 30 s interval emerges as the optimal choice for quantifying neonatal movement. Using it as a reference or pivot, movement values for each computational method and body part are normalized to a baseline of 1, with other intervals expressed in proportional terms (2, 4, and 6 for 60, 120, and 180 s, respectively).

By normalizing to the 30 s interval, Figure 4 provides a standardized comparison between computational methods, body regions, and time intervals. Importantly, the consistent proportionality validates that the shortest interval (30 s) reliably captures meaningful movement differences while balancing accuracy and computational efficiency. This makes it particularly suitable for real-time movement quantification in environments such as the NICUs, where continuous and timely monitoring is essential.

### 3.2. RQ2-Identifying Optimal Methods for Movement Quantification

Based on the findings from RQ1, the optimal interval among the four considered is 30 s. This interval was applied to the study subjects across nine body regions using the methods described in Section 2.4. The results of these experiments are presented in Table 2, where each cell indicates the percentage change in movement—positive values represent an increase, while negative values indicate a decrease—between the subjects’ movement levels near their admission to the NICU and those close to their hospital discharge. For instance, as shown in Table 2, the first row and first column (Euclidean and whole body) indicate that, on average, preterm neonates exhibited 86.70% more movement near hospital discharge compared to their movement levels around the time of admission, based on this method and considering the entire body.

Table 2 includes an Average column, which represents the mean value of all body parts (excluding whole body) for each computational method. Furthermore, the coefficient of variation (CV) is also reported (Coef. Var. column), which is a valid indicator for assessing relative variability, as it normalizes the standard deviation relative to the mean, allowing comparisons across different scales of movement.

The CV is calculated as,

(19) $$CV=\frac{\sigma }{\mu }\times 100$$

where σ is the standard deviation, and μ is the mean of the movement values for each computational method.

The data described in Table 2 provides insights into the effectiveness of each computational method in detecting movement variations and their corresponding reliability:

- Across all computational methods, neonatal movement consistently increased near the discharge date compared to admission, with values ranging from 55.38% (Lucas–Kanade) to 127.01% (Farnebäck).
- The Farnebäck method demonstrated the highest overall movement increase (127.01%) but also exhibited the greatest variability, with a CV of 87.34%. This highlights its sensitivity to movement variations but raises concerns about its reliability for consistent analysis. Similarly, the Lucas–Kanade method reported the lowest percentage increase in movement (55.38%) and a high CV of 26.45%. These results suggest that while Lucas–Kanade is less sensitive to detecting movement variations, its elevated variability further underscores its inconsistencies, making it less suitable for precise neonatal movement quantification. That is, one method underestimates the amount of movement (Lucas–Kanade), while the other overestimates it (Farnebäck), with disproportionate effects on certain body parts, showing increases of 1000% (left arm) and 2000% (right arm). Conveniently, the two methods yielding the worst results are optical flow methods, indicating that they are the least reliable for determining movement quantity in preterm infants. Given their high variability and inconsistent performance, these two optical flow methods (Lucas–Kanade and Farnebäck) were excluded from further analyses related to RQ3.
- Chebyshev Distance demonstrated the lowest variability, with a coefficient of variation (CV) of 5.46%, highlighting its stability and precision. Additionally, the Minkowski and Mahalanobis distance methods demonstrated substantial increases in movement coupled with low variability, making these methods the most robust and reliable options for neonatal movement quantification.
- The remaining distance-based methods, not specifically highlighted, demonstrated moderate performance in terms of both movement increase and variability. While they did not stand out as the most robust or reliable options, neither did they exhibit the highest levels of inconsistency, positioning them as intermediate solutions for neonatal movement quantification.

These findings highlight the variability in computational model performance, emphasizing the need to balance movement detection sensitivity with low variability to ensure reliability. While some computational methods demonstrate robust sensitivity and consistency, others involve trade-offs between accuracy and variability, reinforcing the importance of careful selection based on the specific requirements of neonatal movement quantification.

Given that most techniques produce comparable movement quantification results, an additional differentiating factor is needed. To further refine the selection of the most effective method, we assess computational efficiency by analyzing the average execution time per approach for both individual body parts and the whole body.

**Figure 5:**
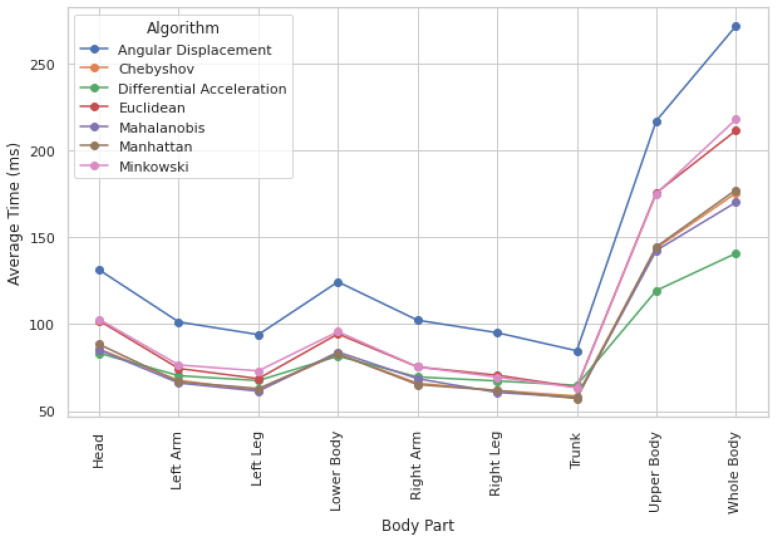
Computational load by method and body region.

Computation times across methods show only slight variations, indicating that execution time is not a critical constraint. While processing the whole body requires the highest computational load (see Figure 5), execution times remain within the millisecond range, making these differences negligible. Additionally, computation times are fairly consistent across different body parts, meaning they have little influence on method selection.

Given the negligible differences in computation time, accuracy and reliability in movement quantification should be the primary factors in method selection. However, when two methods demonstrate similar reliability, opting for the one with a lower computational load can provide an efficiency advantage. Among the evaluated methods, Differential Acceleration is the fastest, followed closely by Chebyshev.

### 3.3. RQ3: Evaluating Whole-Body vs. Targeted Limb Data for Neonatal Movement Quantification

**Figure 6:**
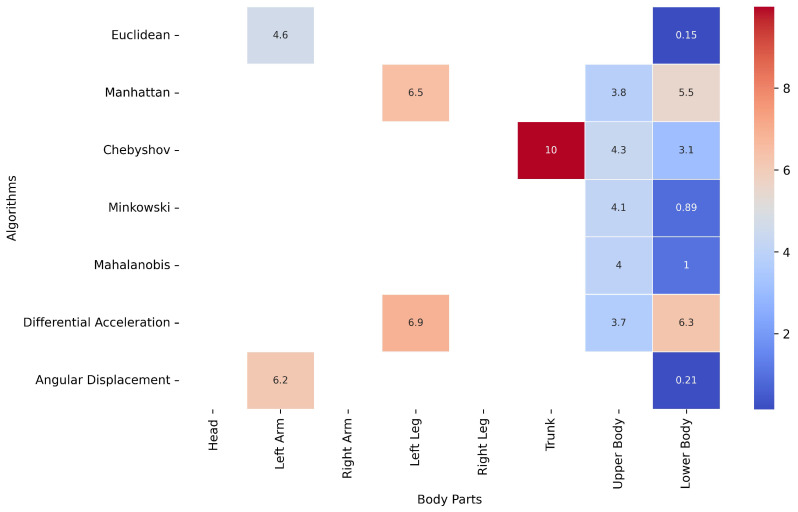
Heatmap of body part movement variation as a percentage of whole-body movement.

Figure 6 presents a heatmap illustrating, for each method, the body part whose movement data closely approximate the movement quantified for the whole body. The percentage values indicate the level of similarity, with a 10% margin of tolerance defining acceptable approximation. In this context, if the difference between the whole-body movement and the movement of a specific body part remains below 10%, that body part can be deemed a reasonable proxy for the whole body.

This analysis reveals that certain body parts, such as the lower body, consistently yield movement data comparable to whole-body results across multiple computational methods, particularly Euclidean (0.15%), Minkowski (0.89%), and Angular Displacement (0.21%) distance metrics.

Additionally, the upper body also closely approximated whole-body movement in some cases, with values around 3.7% (Differential Acceleration) and 3.8% (Manhattan).

Although head, right-arm, and right-leg movement data were analyzed across all methods, they did not meet the similarity threshold in most cases. This suggests that these body parts may not provide reliable approximations of whole-body movement.

The Euclidean method with the lower body data emerges as the most effective combination, as it exhibits the highest level of similarity to whole-body movement within the defined tolerance margin. However, it is important to consider that, according to RQ2, the Euclidean distance exhibited a higher coefficient of variation (15.14%) compared to other methods identified as more suitable, such as Chebyshev, Minkowski, and Mahalanobis.

Among these three, and based on Figure 6, applying Minkowski and Mahalanobis for movement quantification—while using only the landmarks related to the lower body—yields results very similar to those obtained when analyzing the entire body, with a variation of 0.89% for Minkowski and 1% for Mahalanobis.

Therefore, although the Euclidean distance has a relatively low margin of error, Minkowski and Mahalanobis would be more accurate and recommended options for movement quantification.

Using specific body parts instead of analyzing the entire body for movement quantification—while still obtaining comparable results—is particularly beneficial in NICU environments. In these settings, certain body segments may be covered by blankets, or environmental factors such as lighting conditions, neonatal positioning, or medical staff interventions may limit data collection to only some body parts. However, with the appropriate algorithm, it is possible to achieve movement quantification as reliable as using the whole body, making this approach more flexible and practical for clinical applications.

## 4. Discussion

Our research represents a preliminary but significant advancement in preterm infant care, demonstrating that movement quantity can serve as a new clinical indicator for assessing neonatal development. While other developmental parameters, such as weight, size, and physiological metrics, can be objectively quantified, neonatal movement has traditionally been a subjective measure. Our study transforms this subjectivity into objectivity by leveraging computer vision for contactless body data acquisition, combined with advanced computational methods for precise analysis.

In this research, we have successfully analyzed a substantial dataset comprising 800,000 positional data points collected from 10 preterm infants. This dataset enabled a comprehensive examination of movement patterns, revealing a significant correlation between the amount of movement and developmental outcomes. Specifically, it was observed that, in general, all the neonates in the study increased their movement levels when comparing the days close to admission with those near discharge, with the caveat that these infants were discharged in the healthy condition. These findings align with existing literature, which emphasizes the importance of motor activity as an early indicator of neurological health [56,57]. The observed increase in movement levels near discharge suggests that automated motion quantification could serve as one of the indicators for monitoring developmental progress in preterm infants. By integrating such data-driven assessments into neonatal care, healthcare professionals could gain actionable insights beyond what subjective assessments alone can provide. Rather than providing definitive conclusions, the present study aims to suggest a promising direction for incorporating movement quantity into clinical decision-making frameworks.

This study addresses three core aspects: (1) the minimum time required for reliable movement quantification, (2) the identification of the most suitable computational methods for this population among available techniques, and (3) the evaluation of whether full-body data are necessary or if specific body regions can yield equally precise results.

A key contribution is the comparative analysis of several well-established and widely used computational methods, including both optical flow and distance-based approaches. Although we acknowledge the existence of more recent or advanced methods, such as deep learning or motion energy models, our study intentionally focused on traditional methods that are computationally efficient, well-documented, and frequently used in related domains. This choice ensures methodological clarity and broad applicability while laying the groundwork for future comparisons.

Our results indicate that optical flow methods (Lucas–Kanade, Farnebäck) are not suitable for this population group, as they either underestimate or overestimate movement inconsistently. In contrast, distance-based methods (Euclidean, Manhattan, Differential Acceleration, etc.) yield more consistent results, with a variation of approximately 10% among them.

Among these methods, Chebyshev emerges as the most suitable option when analyzing the entire body, due to its lower coefficient of variance, suggesting greater stability in movement quantification. However, if the focus is on specific body regions, the combination of lower-body data with the Minkowski and Mahalanobis methods provides results closest to those obtained with whole-body analysis.

Beyond computational performance, this study demonstrates that shorter observation intervals, particularly 30 s, are sufficient for reliable movement quantification, as evidenced by the proportional scaling of movement values across time windows. This result is particularly valuable for real-time NICU applications, where rapid movement assessment is necessary for timely clinical interventions. It should be noted that time intervals (and their associated movement levels) are only comparable when the neonate is engaged in the same activity during each observation. Movement quantification across different activities inherently produces different results, and thus comparisons between them may not be valid.

These results underscore the importance of selecting methods that balance computational efficiency and accuracy in neonatal motion quantification. By establishing a robust evaluation framework, this study reinforces the feasibility of advanced computational techniques in neonatal care and lays the foundation for further optimization and clinical integration of these methods.

Another key finding of this study is that targeted body regions, especially the lower and upper body, can serve as effective proxies for whole-body movement, reducing the need for analyzing all body landmarks. This approach enhances computational efficiency by limiting the number of processed features, which is particularly advantageous for optimizing real-time movement quantification. Additionally, reducing the number of analyzed landmarks allows for improved robustness in motion estimation techniques, minimizing noise and reducing sensitivity to minor tracking errors, which can be especially beneficial in clinical applications.

Several previous studies have explored electronic and computer-based approaches to assess neonatal movement. For example, Adde et al. (2018) proposed a computer-based video analysis to quantify general movements in preterm infants, supporting the implementation of GMA criteria in an automated way [58]. Similarly, Parisi et al. (2020) introduced a markerless 3D motion tracking system to evaluate motor development during the first year of life. Compared to these approaches, our method uses only 2D pose tracking based on RGB video (or 3D with depth-based camera), requiring no specialized hardware. This makes it more accessible and suitable for routine clinical environments, while still maintaining the goal of providing objective, quantifiable movement indicators [59].

Finally, it is important to acknowledge that this work constitutes a pilot study based on a small sample of subjects, yet with a large number of structured data points per individual. Given this context, we opted for robust descriptive indicators—such as the coefficient of variation (CV) and relative differences between body regions—rather than inferential statistical tests. For instance, the CV values obtained (5.46 and 6.40 for Chebyshev and Minkowski, respectively) reflect low variability between full-body and regional movement estimates. This choice allowed us to evaluate the internal consistency and technical feasibility of our motion quantification approach. While not intended as a formal clinical validation, these results provide a strong basis for future studies involving larger cohorts and deeper statistical and clinical analyses.

### Limitations

Our work represents a preliminary investigation that provides valuable initial observations on neonatal movement patterns and their potential as indicators of developmental progress. Despite these promising outcomes, it is essential to consider the study’s limitations.

Although the dataset of 10 preterm infants offers valuable insights, it may not fully capture the variability found in broader neonatal populations. Nonetheless, this sample enabled a detailed, high-resolution analysis of motion data collected under ethically appropriate and clinically controlled conditions. Recordings were obtained on specific, unscheduled days throughout the hospital stay, rather than through continuous monitoring, to avoid interfering with medical care and to respect parental consent. The timing of the recordings was determined by the availability of the neonates, and gender balance was not prioritized due to the inherent limitations of the sample size. Still, the findings provide a solid foundation for broader investigations. Future research should aim to expand this preliminary work by including more extensive and demographically diverse cohorts, as well as a control group of term infants, to better validate the methodology and assess the specificity or generalization of the observed movement patterns.

Moreover, a limitation in relation to data capture concerns the need for sufficient visual access to the infant’s body in order to obtain reliable pose data. In clinical settings, neonates may be partially or fully covered by blankets or equipment, which can hinder visibility. In cases of full occlusion, motion quantification is not feasible. However, with partial occlusion—for instance, if the lower body is covered—it is still possible to extract motion data from visible regions like the upper torso. As shown in Table 2, the Minkowski algorithm produced similar values when applied to the full body and to the upper body alone (77.78 vs. 80.99), suggesting that partial visibility can still yield meaningful estimates under appropriate conditions.

From a clinical point of view, one of the main limitations of this study is the absence of direct clinical validation against established neurological assessment tools. In particular, we did not include a comparison with the General Movements Assessment (GMA) developed by Prechtl [24], which is widely used for the early detection of neurodevelopmental disorders in preterm and term infants. While this work focused on demonstrating the technical feasibility and internal consistency of automated motion quantification, future studies should integrate concurrent GMA scoring to evaluate the clinical relevance and diagnostic value of the proposed approach.

Finally, a further analytical limitation of this study concerns the choice of computational methods. Our literature review revealed a broad variety of techniques used to quantify movement across diverse populations, such as elderly individuals undergoing rehabilitation and athletes. Some of these methods are well-established—like Euclidean distance and Cosine Similarity—while others are more tailored or population-specific. Given the wide range of available approaches, we deliberately focused on the most frequently applied and validated techniques identified in the literature, which led to the selection of the nine methods analyzed in this study. In future work, we aim to expand upon this foundation by incorporating more recent and data-driven strategies, including artificial intelligence-based methods to explore their potential benefits.

Despite these limitations, and in light of the results, we firmly believe that this study represents a significant advancement in the objective quantification of neonatal movement as a valid medical indicator of preterm infants’ progression. Furthermore, the fact that body movement data can be acquired contactlessly using computer vision techniques underscores the practical impact of this research, paving the way for future developments in computational methods and dedicated movement quantification devices.

With further refinement and validation, these technologies have the potential to revolutionize neonatal care, providing timely, non-invasive, and precise tools for monitoring the health and development of this vulnerable population. The ability to perform reliable, efficient, and targeted analyses positions these approaches as essential components in the next generation of neonatal care solutions.

## 5. Conclusions

In this study, we demonstrated that objective movement quantification in preterm infants is feasible using computer vision techniques combined with computational methods for contactless data capture. We investigated three core aspects: the minimum required observation time, the most suitable computational method, and whether full-body analysis is necessary.

The results showed that 30 s intervals are sufficient for consistent estimation and that distance-based methods (e.g., Chebyshev, Minkowski) yield more reliable outcomes than optical flow, with coefficients of variation under 10%. Notably, analysis of specific body regions (such as the lower body) can yield results comparable to whole-body tracking, enabling computational efficiency without compromising accuracy. Among these, the Chebyshev and Minkowski distances proved to be the most accurate when analyzing a 30 s interval and utilizing whole-body data, with coefficients of variation of 5.46% and 6.40%, respectively, without a significant increase in computational cost compared to other methods. These findings suggest that automated motion quantification could serve as a complementary clinical indicator alongside traditional metrics, such as weight and length, offering reproducible, non-invasive insights into neonatal development.

As future work, we aim to continue expanding the dataset to include a larger and more diverse cohort of preterm infants, enabling more robust statistical and clinical validation. Additionally, an outcome of this research, a first version of the Python library—PyBodyTrack—is already available on PyPI (https://pypi.org/project/PyBodyTrack/ (accessed on 27 June 2025)) and GitHub (https://github.com/bihut/PyBodyTrack (accessed on 27 June 2025)). This tool allows researchers to input frames or videos, select quantification methods, define time intervals, and compute motion metrics automatically. We plan to expand its functionalities progressively to support broader use cases and population groups.

## Figures and Tables

**Table 1:** Medical characteristics.

Variable	M (SD)	Range
Gestational Age (Weeks)	30.1 (1.96)	27–33
Size (cms)		
at Birth	39.7 (3.30)	32–45
at Discharge	44.8 (2.48)	42–48
Head Circumference (cms)		
at Birth	28.15 (2.38)	25–32
at Discharge	32.75 (1.83)	31–35
Weight (gr)		
at Birth	1250.50 (282.74)	850–1860
at Discharge	2515.71 (825.13)	1805–4200
APGAR Score		
1 min	6.90 (1.37)	5–9
5 min	8.30 (1.15)	7–10
CRIB (Clinical Risk Index for Babies)	0.71 (0.95)	0–2

**Table 2:** Average percentage increase in preterm infants obtained using data sampled at 30 s intervals near NICU admission and discharge, across body parts and methods.

Method	Whole Body	Head	Left Arm	Right Arm	Left Leg	Right Leg	Trunk	Upper Body	Lower Body	Average	Coef. Var.
Euclidean	86.70	118.16	82.69	141.76	77.35	105.28	106.78	98.68	86.56	102.15	15.14
Manhattan	76.67	93.14	67.26	102.90	71.71	92.30	89.02	79.60	80.88	84.60	9.38
Chebyshev	78.57	100.59	64.25	102.81	63.06	89.61	86.42	81.98	76.14	83.10	5.46
Minkowski	77.78	98.40	64.91	101.45	64.72	89.87	87.39	80.99	77.09	83.10	6.40
Mahalanobis	77.34	96.66	65.56	101.56	66.77	90.47	87.95	80.45	78.14	83.44	7.31
Differential Acceleration	76.12	92.33	66.44	104.22	70.89	92.77	89.76	78.91	80.90	84.52	9.94
Angular Displacement	84.40	122.48	79.19	139.48	71.72	106.20	104.39	95.36	84.58	100.42	15.95
Lucas–Kanade	55.38	102.35	67.52	76.55	67.98	90.91	67.75	55.35	74.00	75.29	26.45
Farneback	127.01	101.50	1054.57	2304.37	953.76	1237.19	240.27	440.29	794.18	1003.51	87.34

## Data Availability

All datasets used in this study were preprocessed and structured using the NRP platform before being uploaded to NeoVault (https://conversational.ugr.es/neovault—URL (accessed on 27 June 2025)), an open-access data repository. Cleaned postural, physiological, and medical data can be accessed via a user-friendly web interface or through a RESTful API compliant with the OpenAPI standard. The processed data are available in CSV format for download and analysis. Additionally, the complete set of raw postural data is publicly hosted at https://github.com/bihut/neovault-database (accessed on 27 June 2025). Researchers are encouraged to cite the NeoVault database appropriately when using these data in their work.

## Abbreviations

The following abbreviations are used in this manuscript:

NICU	Neonatal Intensive Care Unit
AI	Artificial Intelligence
NRP	Neonates Recording Platform
CV	Coefficient of Variation
JSON	JavaScript Object Notation
CSV	Comma Separated Values
